# *Kaempferia parviflora* Rhizome Extract Inhibits Glutamate-Induced Toxicity in HT-22 Mouse Hippocampal Neuronal Cells and Extends Longevity in *Caenorhabditis elegans*

**DOI:** 10.3390/biology10040264

**Published:** 2021-03-26

**Authors:** Aunchalee Tonsomboon, Mani Iyer Prasanth, Waluga Plaingam, Tewin Tencomnao

**Affiliations:** 1 Interdisciplinary Program of Biomedical Sciences, Graduate School, Chulalongkorn University, Bangkok 10330, Thailand; jim_aun@hotmail.com; 2 Natural Products for Neuroprotection and Anti-Ageing Research Unit, Chulalongkorn University, Bangkok 10330, Thailand; prasanth.m.iyer@gmail.com; 3 Department of Clinical Chemistry, Faculty of Allied Health Sciences, Chulalongkorn University, Bangkok 10330, Thailand; 4 College of Oriental Medicine, Rangsit University, 52/347 Muang Ake, Paholyothin Road, Lakhok, Pathum Thani 12000, Thailand; waluga.p@rsu.ac.th

**Keywords:** *Kaempferia parviflora* rhizome extract, glutamate toxicity, HT-22 mouse hippocampal neuronal cells, *Caenorhabditis elegans*

## Abstract

**Simple Summary:**

In aging societies, age-associated diseases are recognized to diminish people’s quality of life. *Kaempferia parviflora* Wall. ex Baker or “Kra-chai-dam” is a medicinal plant that is known to exhibit numerous pharmacological effects such as anti-inflammation, antimicrobial, and sexual-enhancing activity, and has been used as a nerve tonic. Due to its known neuropharmacological activities, we hypothesize that its rhizome extract might possess both neuroprotective and longevity-enhancing properties. We found that the extract protected glutamate-induced cytotoxicity of mouse hippocampal HT-22 neuronal cells, thus reducing apoptotic cell death. Concomitantly, cells had lower levels of intracellular reactive oxygen species, and changes in the expression of related proteins were found to support the molecular neuroprotection. Furthermore, we found the extract could extend the lifespan of *Caenorhabditis elegans*. Overall, our study proved that the *K. parviflora* rhizome extract possessed neuroprotective and longevity-enhancing properties.

**Abstract:**

*Kaempferia parviflora* Wall. ex Baker (KP) or “Kra-chai-dam” has been shown to exhibit several pharmacological effects including anti-inflammation, antimicrobial, and sexual-enhancing activity. The objectives of this study included an investigation of the effect of KP rhizome extract against glutamate-induced toxicity in mouse hippocampal HT-22 neuronal cells, determination of the underlying mechanism of neuroprotection, and an evaluation of the effect of KP extract on the longevity of *Caenorhabditis elegans*. HT-22 cells were co-treated with glutamate (5 mM) and KP extract (25, 50, and 75 μg/mL) for 14 h. Cell viability, intracellular reactive oxygen species (ROS) assay, fluorescence-activated cell sorting (FACS) analysis, and Western blotting were performed. The longevity effect of KP extract on *C. elegans* was studied by lifespan measurement. In HT-22 cells, co-treatment of glutamate with KP extract significantly inhibited glutamate-mediated cytotoxicity and decreased intracellular ROS production. Additionally, the glutamate-induced apoptosis and apoptotic-inducing factor (AIF) translocation were blocked by KP extract co-treatment. Western blot analysis also demonstrated that KP extract significantly diminished extracellular signal-regulated kinase (ERK) phosphorylation induced by glutamate, and brain-derived neurotrophic factor (BDNF) was recovered to the control. Moreover, this KP extract treatment prolonged the lifespan of *C. elegans*. Altogether, this study suggested that KP extract possesses both neuroprotective and longevity-inducing properties, thus serving as a promising candidate for development of innovative health products.

## 1. Introduction

Aging is a potent risk factor related to many diseases and conditions that limit lifespan including cancers, cardiovascular diseases, and neurodegenerative disorders [1]. The critical change during aging is the loss of irreplaceable cells, mostly in the skeletal muscles, heart, and brain. Alzheimer’s disease (AD) is the most common neurodegenerative disease that causes dementia among the elderly. AD is the sixth leading cause of death for all ages and the fifth leading cause of death in the USA, for those 65 years of age or older [2]. According to the latest WHO data reported in 2017, 3.82% of the total deaths in Thailand was caused by AD and dementia, and AD is now ranked as the tenth leading cause of death [3]. 

It is well known that glutamate is the major excitatory neurotransmitter in the nervous system, but excessive extracellular glutamate concentration can cause excitotoxicity which is a toxic process [4,5], leading to an excessive amount of reactive oxygen species (ROS) and reactive nitrogen species (RNS) in neuronal cells [6]. High ROS might induce oxidative stress, leading to oxidative damage and accelerated aging and cell death [7,8,9]. Consistently, in some model organisms, such as worm, fly, and mouse, there is evidence that reduction in oxidative damage can extend lifespan [10,11]. The ROS produced in the cells have been involved in the activation of numerous cellular signaling pathways and transcription factors including phosphoinositide 3-kinase (PI3K)/Akt, mitogen-activated protein kinase (MAPK), nuclear factor (erythroid-derived 2)-like 2 (Nrf2)/Kelch like ECH-associated protein 1 (Keap1), nuclear factor κB (NF-κB), and the tumor suppressor p53 that can activate cell survival and/or cell death processes including apoptosis [12]. Under oxidative stress, a high level of intracellular ROS promotes the split of Nrf2 and Keap1 via the activation of kinases, such as protein kinase C (PKC), MAPK, PI3Ks, and protein kinase-like endoplasmic reticulum kinase (p-ERK) that phosphorylate Nrf2. Then, the activated Nrf2 is translocated into the nucleus where it binds to the antioxidant responsive element (ARE), and the following ARE-Nrf2 binding enhances the glutathione synthetase (GSS) expression and GSH synthesis for prevention of oxidative stress. Moreover, the receptors of epidermal growth factor (EGF) and platelet-derived growth factor (PDGF) have been shown to be activated by ROS, even in the absence of ligand binding, which can stimulate Ras and the subsequent activation of the extracellular signal-regulated kinase (ERK) pathway [12,13,14,15].

Although many therapies and drugs have been approved for the treatment of AD, they are still not very effective, overpriced, and may cause adverse effect [16]. Recently, herbal medicines have been becoming a popular alternative medication for the remedies of various diseases [17,18]. *Kaempferia parviflora* Wall. ex Baker (KP), also called black ginger or “Kra-chai-dam” in Thai, belongs to the family Zingiberaceae. Certain previous studies have reported the beneficial impacts of KP such as anti-inflammatory, anti-allergic, anticholinesterase, adaptogenic, and anti-obesity effects [19]. A study has also demonstrated that the crude ethanol extract of KP and its compound (5-hydroxy-3,7,3, 4′-tetramethoxyflavone) can inhibit nitric oxide (NO) production in RAW 264.7 cells as well [20]. The effects of KP on neuropharmacological and neuroprotective activities have been examined using two-dimensional gel electrophoresis. The result showed that the ethanol extract of KP can upregulate the expression of glial fibrillary acidic protein (GFAP) and dihydropyrimidinase-related protein 2 (Dpysl2) which are related to anti-oxidative activity and microtubule formation, respectively. Moreover, KP extract also increased the production of norepinephrine (NE), serotonin (5-HT), and dopamine (DA) in the hippocampus of Sprague–Dawley (SD) rats [21]. Moreover, 5,7-dimethoxyflavone is a major active compound of KP [22] that has exhibited remarkable inhibitory activity similar to acetylcholinesterase (AChE) and butyryl-cholinesterase [23,24]. In this study, we aimed to investigate the biological effect of KP rhizome extract against glutamate-induced toxicity in mouse hippocampal HT-22 neuronal cells and to also determine the underlying mechanism of neuroprotection. Furthermore, the effect of KP on the longevity of *Caenorhabditis elegans* was also evaluated. We hypothesized that the KP rhizome extract can protect against the neurotoxicity which is induced by glutamate in HT-22 cells and extend the lifespan of *C. elegans*.

## 2. Materials and Methods

### 2.1. Chemicals and Reagents

Chemicals and reagents were purchased as follows: L-glutamic acid, 2,2′-azino-bis(3-ethylbenzothiazoline-6-sulfonic acid) diammonium salt (ABTS), 2,2-diphenyl-1-picrylhydrazyl (DPPH), quercetin, curcumin, dimethyl sulfoxide (DMSO), Dulbecco’s modified eagle’s medium (DMEM), fetal bovine serum (FBS), and N-acetyl cysteine (NAC) from Sigma–Aldrich (St. Louis, MO, USA); 10× penicillin-streptomycin from Hyclone (UT, USA); Bradford reagent from Bio-Rad (Hercules, CA, USA); 3-(4,5-dimethylthiazol-2-yl)- 2,5-diphenyl tetrazolium bromide (MTT) from Bio Basic (Markham, ON, Canada); gallic acid from TCI America (Portland, OR, USA); L-ascorbic acid from Calbiochem (San Diego, CA, USA); 2′, 7′-dichlorodihydro fluorescein diacetate (H_2_DCFDA) from Molecular Probes (Eugene, OR, USA); trypsin-EDTA (0.25%) and penicillin/streptomycin solution from Gibco (Waltham, MA, USA); primary antibodies against p44/42 kinase (ERK), phospho-p44/42 (p-ERK), apoptotic-inducing factor (AIF), β-actin, Lamin B1, and goat anti-rabbit secondary antibodies from Cell Signaling Technology (Danvers, MA, USA); primary antibodies against brain-derived neurotrophic factor (BDNF) from Abcam (Cambridge, UK); the annexin V- Fluorescein isothiocyanate (FITC) apoptosis detection kit from BioLegend (San Diego, CA, USA); and the NE-PER nuclear and cytoplasmic extraction reagents from Thermo Scientific (Rockford, IL, USA).

### 2.2. Plant Extraction

KP was collected from the Loei Province in Northeast Thailand. The rhizomes were shade-dried and agitated by using a blender, and the powder was extracted with 95% ethanol by using the Soxhlet extractor for 24 h. The extract was dissolved in dimethyl sulfoxide (DMSO) to a final concentration of 100 mg/mL as the stock solution and stored at −20 °C until needed for the experiments.

### 2.3. Cell Line

Mouse hippocampal HT-22 cells (a generous gift from Professor David Schubert, Salk Institute, San Diego, CA, USA) were cultured in DMEM medium (Hyclone, Logan, UT, USA), supplemented with 10% (*v*/*v*) fetal bovine serum and 1% penicillin/streptomycin, in a humidified atmosphere containing 5% CO_2_ at 37 °C. The cells were seeded and incubated overnight prior to treatment with glutamate alone or in co-treatment with various concentrations of KP extract for the next experiment.

### 2.4. 3-(4,5-Dimethylthiazol-2-yl)-2,5-diphenyltetrazolium Bromide (MTT) Assay

Cultured HT-22 cells were plated in 96-well plates at a density of 7 × 10^3^ cells/well, and grown for 24 h in a humidified atmosphere containing 5% CO_2_ at 37 °C. The following day, cultured media was removed, and then cells were treated with various concentrations of KP extract and 5 mM glutamate. NAC 0.25 mM and 1% of DMSO were also used as positive and negative controls, respectively. After incubating for 14 h, the MTT solution was added to culture medium at a final concentration of 0.5 mg/mL and left in the dark for 3 h at 37 °C. Absorbance was measured using a microplate reader (EnSpire^®^ Multimode Plate Reader (PerkinElmer, Waltham, MA, USA)) at 550 nm for the determination of cell viability.

### 2.5. Reactive Oxygen Species (ROS) Assay

The ROS scavenging ability of KP extract was evaluated using the 2′, 7′-dichlorodihydro fluorescein diacetate (H_2_DCFDA) (general oxidative stress indicator). After 14 h of treatment, HT-22 cells were incubated with 10 μM of H_2_DCFDA for 30 min at 37 °C, followed by washing three times with phosphate buffer saline (PBS). The fluorescence intensity (excitation = 485 nm and emission = 535 nm) was measured using an EnSpire^®^ Multimode Plate Reader (PerkinElmer) and the photographs were obtained using an Axio Observer A1 fluorescence microscope (Carl Zeiss, Jena, Germany). Data were collected and analyzed as the percentage of fluorescence intensity of treated cells relative to untreated control and glutamate control.

### 2.6. Apoptosis Assay by Flow Cytometer

HT-22 cells (1 × 10^5^ cells) cultured in a 12-well plate were co-treated with 5 mM of glutamate and KP extract (50 and 75 µg/mL) for 14 h. For fluorescence-activated cell sorting (FACS) analysis, cells were harvested, washed, and stained with annexin V/PI solution for 15 min in the dark. Live and dead cells were determined by using a BD FACSCalibur™ flow cytometer (BD Bioscience, Heidelberg, Germany). Data were collected from at least 10,000 cells per group and results are shown as the percentage of apoptotic cells.

### 2.7. Protein Expression by Western Blotting

After 14 h of treatment, treated cells were collected and lysed using a lysis buffer containing 20 mM Tris-HCl (pH 7.5) 1% Triton X, 150 mM sodium chloride, 10% glycerol 1 mM sodium orthovanadate, 50 mM sodium fluoride, 100 mM phenylmethylsulfonyl fluoride, and commercial protease inhibitor cocktail (Roche Molecular Biochemicals). Then, the protein lysates were stored at −80 °C until use. 

For nuclear and cytoplasmic extraction, the whole treated cell was trypsinized, and cytoplasmic and nuclear fractions were extracted using the NE-PER nuclear and cytoplasmic extraction reagents (Thermo Scientific), in accordance with the manufacture’s protocol. 

Total protein concentrations were quantified by the Bradford assay, and mixed with Laemmli loading buffer and incubated at 95 °C for 10 min before loading into a 12% sodium dodecyl sulfate (SDS) polyacrylamide gel. Then, the protein was transferred to PVDF membranes and blocked in 5% blocking buffer (BioRad, Hercules, CA, USA) in TBST (25 mM Tris-HCl, pH 7.5, 125 mM NaCl, and 0.1% Tween 20). After blocking for 90 min, the membranes were washed 3 times with TBST, and then probed with the following primary antibodies: ERK (1:2000), p-ERK (1:2000), BDNF (1:1000), AIF (1:1000), lamin B1 (1:1000), and β-actin (1:10,000) at 4 °C overnight. The following day, membranes were washed thrice with TBST for 10 min, and incubated with HRP-conjugated secondary antibodies (1:15,000) at room temperature for 1 h. Subsequently, the bands were exposed to X-ray film with the chemiluminescence detection system (ECL™ Select Western Blotting Detection Reagent, GE Healthcare, Piscataway, NJ, USA) and quantified by Image J software. 

### 2.8. Nuclear Apoptotic-Inducing Factor (AIF) Translocation by Immunofluorescent Staining

The treated cells were fixed with 4% (*w*/*v*) paraformaldehyde in PBS for 10 min, washed thrice with PBS, and permeabilized in 0.1% (*w*/*v*) Triton X-100 in PBS for 10 min at RT, and then blocked with 5% bovine serum albumin (BSA) in PBS for 1 h at room temperature. The cells were washed with PBS and probed with primary antibodies against AIF (1:200) at 4 °C overnight. Alexa Fluor 488-conjugated goat anti-rabbit (1:2000) was added to the cells and incubated, in the dark, for 1 h at room temperature, and then washed with PBS and counterstained with Hoechst (1:2000), in the dark, for 10 min at room temperature. The images were captured using an LSM 700 confocal laser scanning microscope (Carl Zeiss, Jena, Germany).

### 2.9. Antioxidant Determination 

#### 2.9.1. Radical Scavenging Activity Assays

The free radical scavenging activity of KP extract was determined, as previously described [25]. The DPPH• solution was diluted in absolute ethanol at a concentration of 0.2 mg/mL and the ABTS• + solution was freshly diluted with absolute ethanol to obtain an absorbance of 0.7 to 0.8 at 734 nm. After the addition of 1 part of KP extract and 9 parts of DPPH• or ABTS• + solution (dilution 1:10), the absorbance was measured at 517 nm after 15 min of the initial mixing for DPPH assay and at 734 nm after 30 min of incubation for ABTS assay, using an EnSpire^®^ Multimode Plate Reader (PerkinElmer) with ascorbic acid (vitamin C) as the standard. All the determinations were performed in triplicates. Radical scavenging activity was calculated as the percent inhibition of the radical calculated using the following equation:

% Inhibition = 100 − [(Abs of sample − Abs of blank) × 100/Abs of control]

#### 2.9.2. Total Phenolic Determination

The total phenolic content was determined by the Folin–Ciocalteu method [26], with slight modifications. Briefly, 50 μL of KP extract (1 mg/mL) was mixed with 50 μL of a 10% Folin–Ciocalteu reagent, and then incubated in the dark at room temperature for 20 min, followed by the addition of 50 μL of 7.5% (*w*/*v*) Na_2_CO_3_ solution for neutralization, and then kept in the dark at room temperature for 20 min. The absorbance was measured at 760 nm using an EnSpire^®^ Multimode Plate Reader (PerkinElmer). The total phenolic content was calculated as mg gallic acid equivalent (mM GAE) by using a gallic acid calibration curve.

#### 2.9.3. Total Flavonoid Determination

The total flavonoid content of crude extract was determined by the aluminum chloride colorimetric method [27], with slight modifications. Briefly, 50 μL of KP extract (1 mg/mL) was added into the plate with absolute ethanol to obtain the final volume 200 μL, and then homogeneously mixed with 10 μL of 10% (*v*/*v*) AlCl_3_ solution and 10 μL of 1 M NaOAc solution. After 40 min of incubation period, the absorbance was measured at 415 nm and the total flavonoid content was determined. In this method, quercetin was used to make the standard calibration curve and the result was presented as mg of quercetin equivalent (QE) per g of dry weight plant extract. 

### 2.10. Nematode Strain, Culture Condition, and Lifespan Assay

Wild-type *C. elegans* (Bristol N2) were procured from the *Caenorhabditis* Genetics Center (University of Minnesota, Twin Cities, MN, USA) and cultured in nematode growth medium (NGM) agar plates with *E. coli* OP50 as the food source at 15 °C [28]. The lifespan of wild-type *C. elegans* was measured, as previously described [28]. The nematodes were treated with various concentrations of KP extract ranging from 0 to 900 μg/mL for assessing whether KP extract could extend the *C. elegans* lifespan. In brief, the young adult nematodes were transferred to a 24-well plate with 500 μL of M9 buffer supplemented with the *E. coli* OP50 and various doses of KP extract. The worms were grown at 15 °C. The total number of worms were counted daily and scored as dead when they did not respond to a gentle tap or touch with the platinum loop. All the experiments were performed with at least 10 worms per group and done in 5 independent trails. 

### 2.11. Statistical Analysis

All experiments were performed in at least triplicate and shown as the mean ± SEM. The difference between the treatment groups and control were calculated using one-way ANOVA analysis following the post hoc Tukey HSD test with SPSS software (SPSS, ver. 21.0, Inc., Chicago, IL, USA). For the lifespan assay, a comparison of the survival distributions among different groups was done by a log-rank (Mantel−Cox) test. All statistical analyses were analyzed using SPSS software, and *p*-value < 0.05 indicated statistical significance.

## 3. Results

### 3.1. The Protective Effect of Kaempferia parviflora Wall. ex Baker (KP) Extract on Glutamate-Treated HT-22 Cells

**Figure 1:**
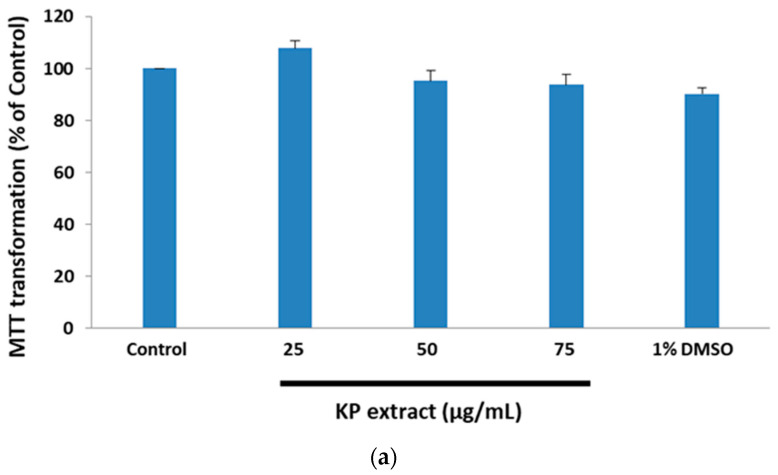

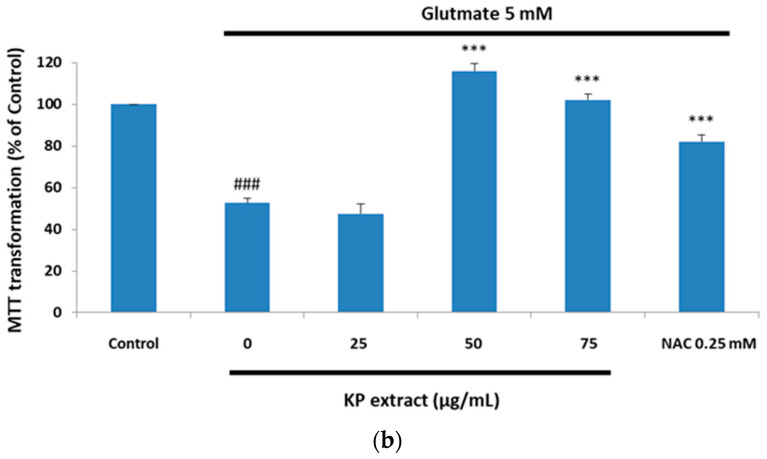
Neuroprotective effect of *Kaempferia parviflora* Wall. ex Baker (KP) extract on glutamate-induced cytotoxicity in HT-22 cells. (**a**) Cells were treated with various concentrations of KP extract (25, 50, and 75 µg/mL) for 14 h; (**b**) cells were exposed to 5 mM of glutamate alone or glutamate in combination with different concentrations of KP extract for 14 h. Cell viability was determined by 3-(4,5-dimethylthiazol-2-yl)- 2,5-diphenyl tetrazolium bromide (MTT) assay. Each bar represents the mean ± SEM from 3 independent experiments per group. ^###^ *p* < 0.001 vs. control and *** *p* < 0.001 vs. the glutamate alone.

KP extract has certain beneficial properties, such as antioxidant and neuroprotective activities [19,22]. Considering this, we investigated the effect of KP extract on glutamate-induced cytotoxicity in HT-22 cells. The cytotoxic effect of KP extract against HT-22 cells was determined by incubating 1 × 10^4^ cells/well in a 96-well plate with various concentrations of KP extract (25, 50, and 75 µg/mL) for 14 h. The percentages of cell viability were determined by MTT assay. The result showed that KP extract did not exhibit cytotoxicity in HT-22 cells as the cell viability was retained above 80% (Figure 1a). To examine the ability of KP extract for neuroprotective effects against glutamate-induced cell death, the HT-22 cells were co-treated with non-cytotoxic concentrations of KP extract and 5 mM of glutamate for 14 h, and 0.25 mM of N-acetylcysteine (NAC) was used as a positive control. The cell viability was measured by MTT assay, wherein 5 mM of glutamate treatment caused the reduction of cell viability to 52.6 ± 2.12%, and KP extract co-treatment at 50 and 75 µg/mL increased the cell viability to 115.97 ± 3.41% and 102.11 ± 2.72%, respectively. There were statistically significant differences in the percentage of viability of glutamate treated HT-22 cells, with or without KP (50 and 75 µg/mL) extract, whereas there was no change in percentage of viability of the HT-22 group that was exposed to 25 µg/mL of KP extract (Figure 1b).

### 3.2. The Inhibitory Effect of KP Extract on Glutamate-Induced ROS Production

**Figure 2:**
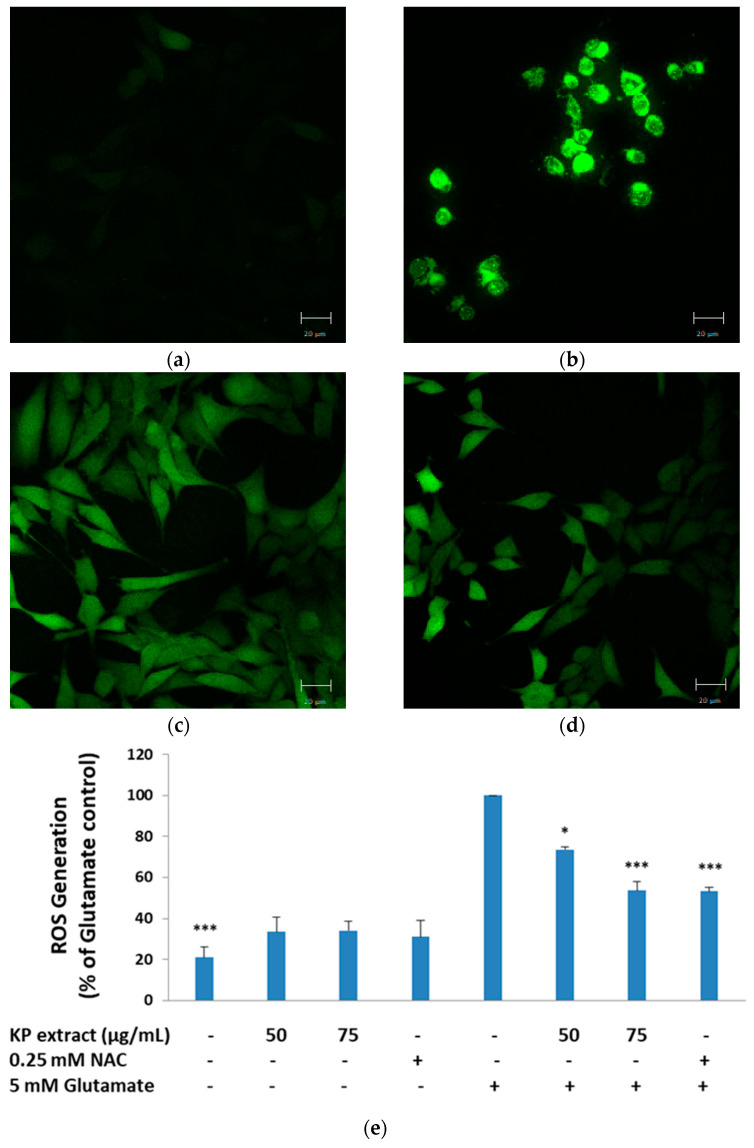
Inhibitory effect of KP extract against glutamate-induced oxidative stress in HT-22 cells. HT-22 cells were treated with 5 mM glutamate alone or co-treatment of glutamate with various concentrations of KP extract for 14 h. The production of intracellular reactive oxygen species (ROS) was stained by 2′, 7′-dichlorodihydro fluorescein diacetate (H_2_DCFDA) and observed under a fluorescence microscope. (**a**) Untreated cells (control); (**b**) 5 mM of glutamate; (**c**) 75 µg/mL of KP extract + 5 mM of glutamate; (**d**) 0.25 mM of N-acetyl cysteine (NAC) + 5 mM of glutamate; (**e**) the quantity of production of intracellular ROS was measured using H_2_DCFDA by the fluorescence microplate reader. All data are shown as the mean ± SEM of at least three independent experiments. * *p* < 0.05 and *** *p* < 0.001 vs. the glutamate treatment alone. The symbol “-” and “+” are defined as absent and present, respectively.

The antioxidant activity of KP extract was evaluated, including DPPH, ABTS radical scavenging activity, and total phenolic and flavonoid contents, as shown in Table 1. In order to measure the intracellular ROS production in response to glutamate (5 mM), HT-22 cells were co-treated with KP extract and glutamate for 14 h, and then stained with H_2_DCFDA. As expected, the results showed that 5 mM of glutamate treatment significantly increased ROS generation (*p* < 0.001), whereas the KP extract co-treatment dramatically reversed this trend, as ROS generation of 50 and 75 µg/mL of KP extract were 73.56 ± 1.37% (*p* < 0.05) and 53.63 ± 4.31% (*p* < 0.001), respectively, which were lower than 5 mM glutamate-treated group (Figure 2a–e). These results showed that co-treatment with the KP extract protected HT-22 cells against glutamate-induced cell oxidative damage.

### 3.3. The Anti-Apoptotic Activity of KP Extract on HT-22 Cells

**Figure 3:**
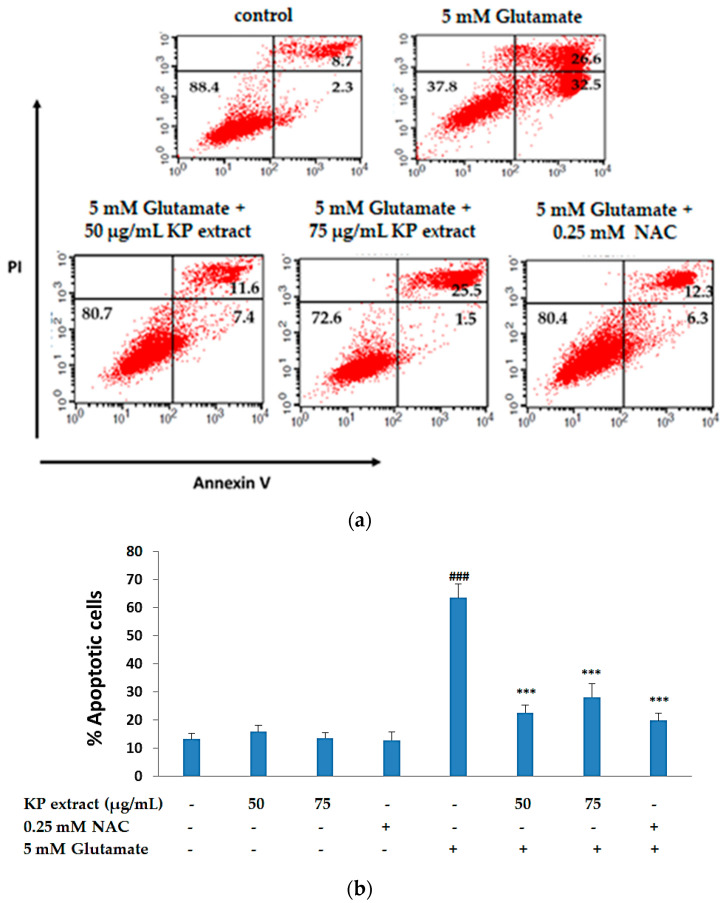
The anti-apoptotic activity of KP extract on HT-22 cells. (**a**) Representative flow cytometry plots using Annexin V-FITC/PI staining in each group including an untreated group (control), 50 and 75 µg/mL of KP extract treatment in the presence or absence of 5 mM of glutamate for 14 h. NAC (0.25 mM) was used as positive control; (**b**) bar graph indicates the percentage of apoptotic cells (PI-/annexin V + stained cell population). Data are presented as the mean ± SEM (n = 3). ^###^ *p* < 0.001 vs. control and *** *p* < 0.001 vs. 5 mM of glutamate. The symbol “-” and “+” are defined as absent and present, respectively.

As previously reported, HT-22 cells treated with glutamate show signs of apoptosis and necrosis in a time-dependent manner [29]. In order to investigate the role of KP extract in glutamate-induced apoptosis in HT-22 cells, the cells were treated with glutamate, with and without KP extract for 14 h and annexin V-FITC/PI staining was done to measure early and late apoptotic cells. After 14 h of HT-22 cell treatment with 5 mM of glutamate, we found an increase in the PI-/annexin V + stained cell population to 63.43 ± 4.94%. In the presence of 50 and 75 µg/mL of KP extract along with glutamate, the PI-/annexin V + stained cell population reduced to 22.49 ± 2.83 and 28.00 ± 4.83, respectively, and co-administration of NAC 0.25 mM significantly reduced apoptotic cell death (*p* < 0.001 for all doses, Figure 3a,b). Thus, the FACS data indicated that KP extract exhibited potent neuroprotective effects against glutamate treatment.

### 3.4. The Effects of KP Extract on Signaling Molecules Associated with Caspase-Independent Apoptotic Pathway

**Figure 4:**
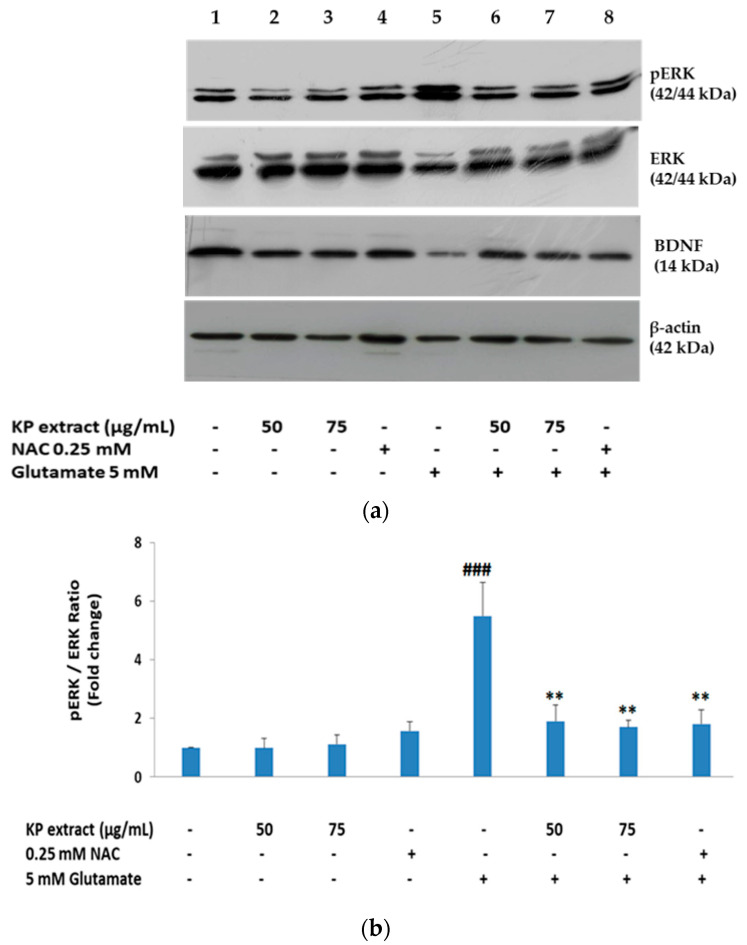

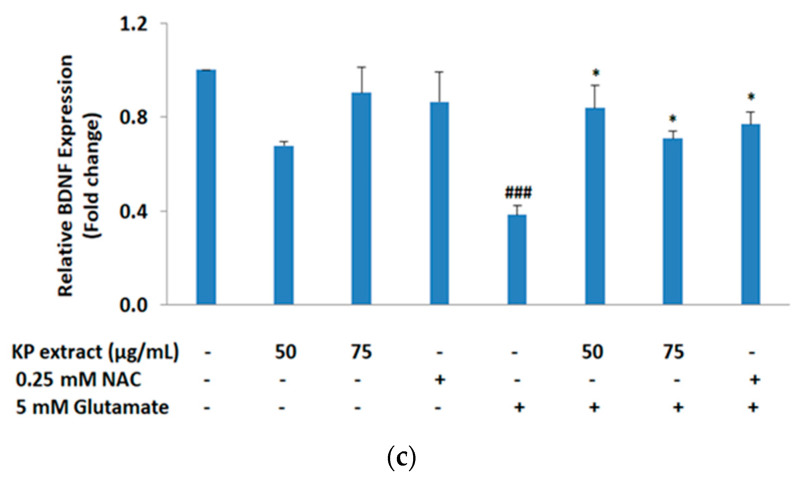
Effect of KP extract on the glutamate-induced phosphorylation of extracellular signal- regulated kinase (ERK) and brain-derived neurotrophic factor (BDNF) expression. (**a**) HT-22 cells were exposed with 50 and 75 μg/mL KP extract in the presence or absence of glutamate (5 mM) for 14 h, NAC (0.25 mM) was used as positive control. Following incubation, the cells were harvested and BDNF, ERK, and protein kinase-like endoplasmic reticulum kinase (p-ERK) levels were investigated by Western blot analysis. The immunoreactive bands were detected using specific antibodies for BDNF, ERK, p-ERK, and β-actin; (**b**,**c**) bars represent the fold-increase in phosphorylation of mitogen-activated protein kinases (MAPKs) and BDNF expression, respectively. All data were normalized to internal control levels and are expressed as the mean ± SEM (n = 3), ^###^ *p* < 0.001 vs. control, * *p* < 0.05 and ** *p* < 0.01 vs. glutamate alone. The symbol “-” and “+” are defined as absent and present, respectively.

As previously reported, MAPKs including p38, ERK, and c-Jun N-terminal kinase (JNK) accomplish crucial roles in apoptotic signal transduction [30]. Accumulation of intracellular ROS activates MAPK signaling, which indicates that ROS is involved in MAPK activation. Therefore, glutamate-mediated oxidative stress causes neuronal cell death through MAPK activation [30,31]. Moreover, as we know that activation of the MAPK/ERK signaling pathways is impacted by BDNF [32], we have characterized the relevance of these signaling molecules including BDNF, ERK, p-ERK, and AIF (cytosolic and nuclear fractions) in KP extract-treated HT-22 cells using Western blot analysis. Glutamate treatment on HT-22 cells for 14 h showed the reduction of mature BDNF expression and also activated the mitogen-activated protein kinase (MAPK) pathway, evidenced by the presence of the phosphorylated form of ERK, whereas co-treatment condition with 50 and 75 µg/mL of KP extract enhanced the mature BDNF expression and inactivation of p-ERK significantly (*p* < 0.05 and *p* < 0.01 for all doses, Figure 4a–c). NAC also showed similar effects to those of KP extract.

**Figure 5:**
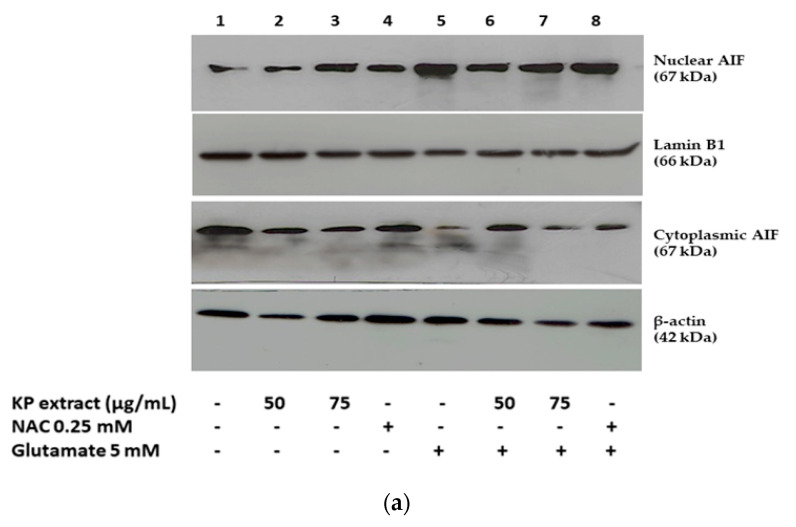

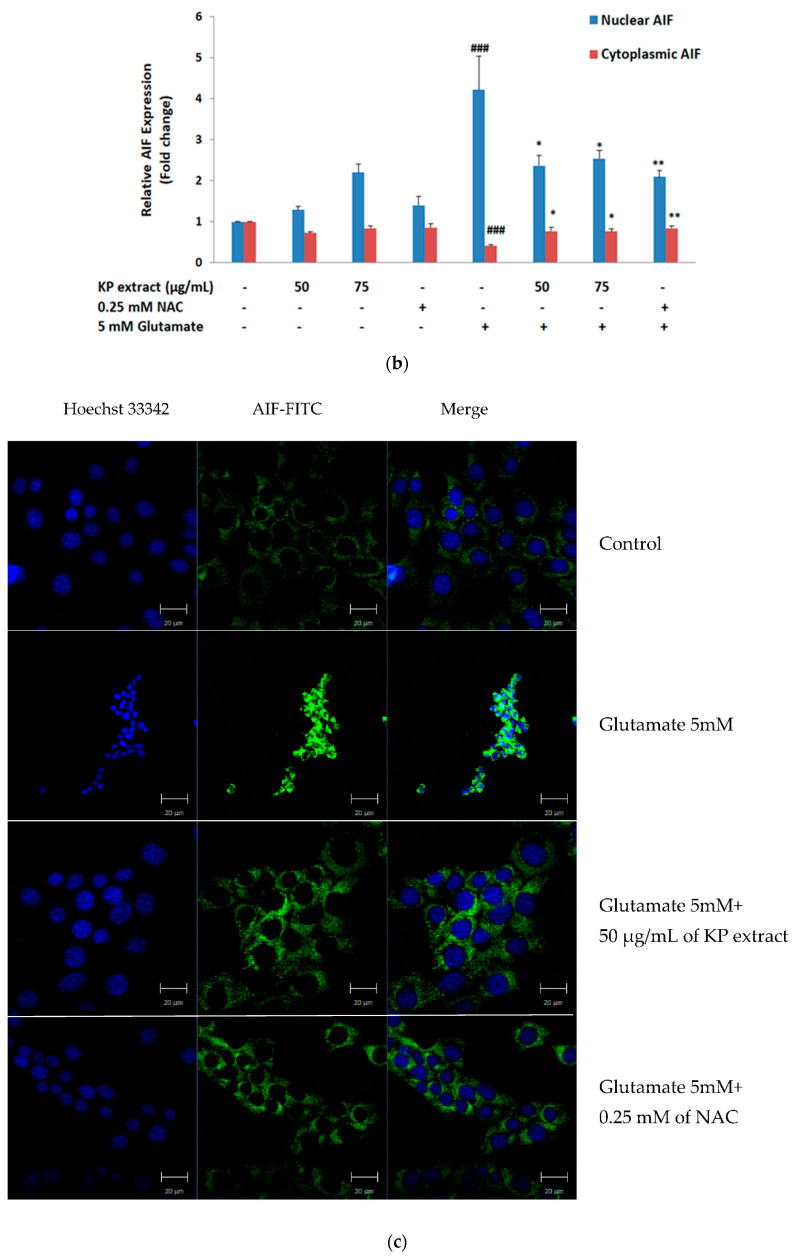
The effect of KP extract against glutamate-mediated cell death involves apoptotic-inducing factor (AIF) translocation to the nucleus. HT-22 cells were exposed with 50 and 75 μg/mL KP extract in the presence or absence of glutamate (5 mM) for 14 h, NAC (0.25 mM) was used as positive control. (**a**) Following incubation, nuclear and cytoplasmic fractionation was performed and all targeted proteins (then, nuclear AIF, cytoplasmic AIF, Lamin B1, and β-actin were analyzed using Western blot analysis; (**b**) bars represent the fold-change of nuclear and cytoplasmic AIF expression. Lamin B1 and β-actin were used as an internal control to normalize protein expression for nuclear extracts and whole cell/cytoplasmic extracts, respectively. All data are expressed as the mean ± SEM (n = 3), ^###^ *p* < 0.001 vs. control, * *p* < 0.05, and ** *p* < 0.01 vs. glutamate alone. The symbol “-” and “+” are defined as absent and present, respectively; (**c**) confocal laser scanning microscope images of AIF immunoreactivity (green) and nuclear Hoechst 33342 staining (dark blue). Co-treatment with KP extract resulting in prevention of nuclear AIF translocation after 14 h of 5 mM of glutamate exposure.

We also examined the antiapoptotic mechanisms of KP extract against glutamate-induced cell death by measuring the AIF translocation by immunofluorescence and Western blotting analysis. Following treatment with 5 mM glutamate for 14 h, the AIF proteins translocate into the nucleus and the results showed the nuclear AIF fraction levels were significantly increased, whereas cytoplasmic AIF decreased. After co-treatment with 50 and 75 µg/mL of KP extract, significant reduction in the translocation of these proteins into the nucleus (Figure 5a,b) was observed. For confocal microscopy, the results were consistent with Western blot analysis (Figure 5c). The finding suggests that the neuroprotective effect of KP extract occur via inhibition of the caspase-independent apoptotic pathway. some Western blot images as representatives showing the expression of Erk, pErk, BDNF, β-actin, nuclear AIF, Lamin B1, cytoplasmic AIF, β-actin were shown in Figure S1.

### 3.5. The Effect of KP Extract on Longevity of Caenorhabditis elegans

**Figure 6:**
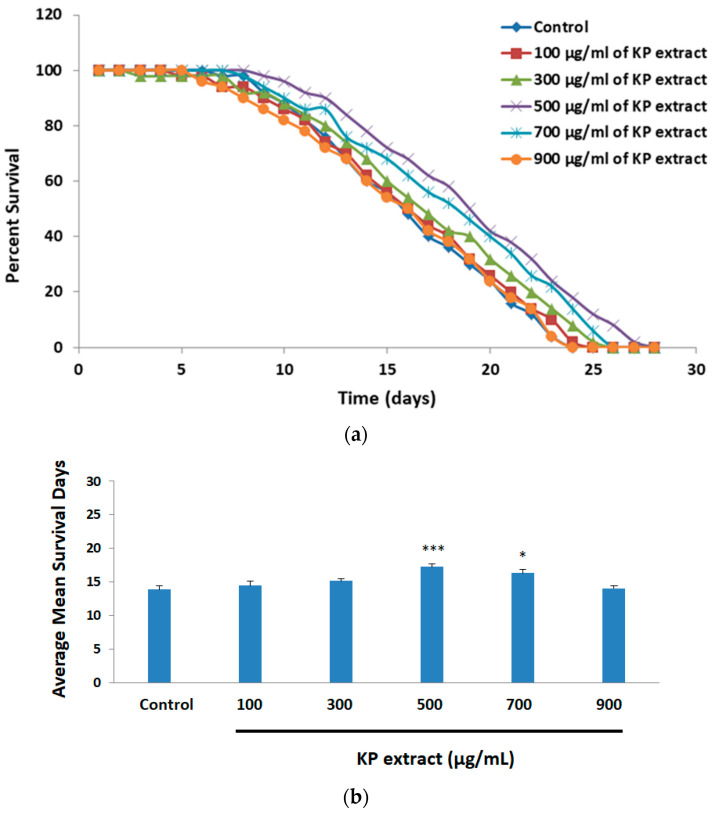
Effect of KP extracts on the lifespan of N2 wild-type *C. elegans*. The nematodes were treated with various concentration of KP extracts (0, 100, 300, 500, 700, and 900 µg/mL) at 15 °C. The survival was counted every day till death. (**a**) Survival curves of KP extracted treated *C. elegans* as compared with the untreated group (control); (**b**) Bars represent the mean lifespan when treated with KP extract. The experiment was performed 5 independent trails and shown as the mean ± SEM, * *p* < 0.05 and *** *p* < 0.001 vs. control.

The N2 wild-type *C. elegans* cultured on various concentrations of KP extracts (100, 300, 500, 700, and 900 μg/mL) were monitored to determine whether KP extract had any effect on the lifespan of *C. elegans*. The survival curves and results are shown in Figure 6a,b. *C. elegans* treated with KP extract at 500 and 700 µg/mL showed extension of lifespan (Figure 6a). Our results revealed that the control group without KP extract had a mean lifespan of 13.86 ± 0.58 days. There was no statistical significance in the lifespan of the *C. elegans* control group and those exposed to KP extract at lower concentrations (100 and 300 μg/mL). However, *C. elegans* treated with KP extract at 500 and 700 μg/mL significantly extended the mean of lifespan to 17.24 ± 0.55 and 16.28 ± 0.42 days, (*p* < 0.001 and *p* < 0.05) (Figure 6b). As data demonstrated above, KP extract could markedly prolong the lifespan of *C. elegans*. Data of KP extract treated *C. elegans* according to lifespan assay were also shown in Table S1. 

## 4. Discussion

It is well known that excessive accumulation of β-amyloid peptide (Aβ) is one of the major mechanisms responsible for neuronal death in AD. Plant-derived flavonoids have shown promise for improving AD, but the overall mechanism of action is still unknown. In some cases of neurodegenerative diseases, it has been demonstrated that the protein aggregation including α-synuclein and amyloid β fibrillogenesis are critical factors for the disease development, similar to transthyretin (TTR) amyloidoses that are protein aggregation diseases associated with peripheral neuropathy. Flavonoids act as antioxidants which are a group of polyphenolic compounds synthesized in plant cells. Certain previous studies have revealed that the flavonoid compounds, especially epicatechin, epigallocatechin gallate (EGCG), luteolin, and myricetin could inhibit Aβ-induced neuronal death. They have also shown that EGCG can bind to TTR and that EGCG inhibits TTR aggregation which involves amyloid fibril formation in vitro and in a cell culture system. In addition, curcumin, a natural occurring polyphenol, which presents structural similarities with thyroxine (T4), can modulate TTR abnormal aggregation and counteract TTR tissue deposition, therefore inhibiting the process of TTR amyloid fibril formation as compared with EGCG [33,34,35,36,37,38]. 

KP is a Thai herbal plant comprised of many flavonoids which are widely used to treat various diseases [39]. Recently, many therapeutic functions of KP have been reported including anti-inflammation, anti-allergic, anti-obesity, anti-gastric ulcer, anti-depressant, anticholinesterase, antimicrobial, aphrodisiac, vasodilation, and antioxidant activities [40]. According to the previous reports by Wongsrikaew et al., 2012 [41] and Mekjaruskul et al., 2013 [42], typical HPLC chromatograms of KP rhizome ethanol extract were found to have 5,7-dimethoxyflavone (DMF), 3,5,7,3′,4′-pentamethoxyflavone (PMF), and 5,7,4′-trimethoxyflavone (TMF) as major phytochemicals. In this study, our KP extract was shown to have DMF as a major chemical constituent as presented in Figure S2. 

A few studies have previously reported the neuropharmacological and neuroprotective effects of KP. Interestingly, the proteomic analysis formerly performed by our laboratory has revealed that the ethanolic KP extract containing DMF as a main chemical constituent upregulates GFAP and DPYSL2, which are involved in the anti-oxidative process and microtubule formation, respectively [21]. Moreover, the neurotransmitters including norepinephrine (NE), serotonin (5-HT), and dopamine (DA) increase in rat hippocampus after KP extract treatment [21]. In addition, preliminary research has noted that KP exhibited acetylcholinesterase inhibitory activity which may be a good choice for AD therapy [24].

Oxidative stress may be one of the main causes of several neurological disorders. Additionally, factors such as excessive glutamate accumulation and activation of glutamatergic neuron enhanced ROS generation, oxidative stress, excitotoxicity, and injury of neurons. The neurotoxicity effect of glutamate is linked to ROS generation through receptor-mediated excitotoxicity or non-receptor mediated oxidative toxicity [43,44] and is involved in neuronal apoptosis including caspase-dependent and independent signaling pathways [45,46].

In this regard, our aim was to investigate the biological activities of antioxidant containing KP extract against glutamate-induced toxicity in mouse hippocampal HT-22 neuronal cells. The HT-22 cells are commonly selected to study the effects of glutamate-induced oxidative stress because these cells do not express the glutamate N-methyl-D-aspartate (NMDA) receptor [47,48], in this way the mechanism of glutamate-induced oxidative stress and cell death mostly occurs via the interruption of the cysteine/glutamate exchange transporter by excessive extracellular glutamate, resulting in a decreased level of intracellular glutathione and ROS accumulation [49]. Unfortunately, the other glutamate toxicity pathway, which is the glutamate receptor-dependent pathway, could not be investigated in our model. 

In our experiments, we compared the neuroprotective capability of KP extract on glutamate-mediated neuronal toxicity in HT-22 cells using MTT assay. About 50% of cell viability remaining after 5 mM glutamate exposure for 14 h which was consistent with a previous finding [25]. Surprisingly, the decline of cell viability by glutamate was significantly recovered when exposed to 50 and 75 µg/mL of KP extract. NAC was used as a positive control. Accordingly, deciding on an optimal dose of NAC to use in our experiment, HT-22 cells were treated with various concentration of NAC (0.125, 0.25, 0.5, and 1 mM) for 14 h. The result indicated that 1 mM of NAC mediated toxicity in HT-22 cells (cell viability less than 80%). At 0.25 and 0.5 mM of NAC, treatment provided the same results (cell viability ≈ 85–90%) (data not shown). Therefore, the optimal dose of 0.25 mM NAC was used as a positive control in all experiments. Nevertheless, when HT-22 cells were treated with KP extract prior to glutamate exposure, significant protection of glutamate-induced toxicity was not found (Figure S3). The effects of various concentrations of KP extract and glutamate on the viability of HT-22 cells were also shown (Figure S4). 

Here, we also examined the inhibitory effect of KP extract on glutamate-induced ROS production using H_2_DCFDA assay. It has been reported that the increased ROS production following glutamate treatment may be related to an elevation in mitochondrial membrane potential (MPP). MPP was reported to gradually increase and reach a peak at 12 h and remain elevated 30% higher than the control even after 24 h of glutamate exposure [50]. Similarly, we found that after 14 h of glutamate exposure, the elevation levels of intracellular ROS in glutamate-treated HT-22 cells were significantly different as compared with untreated control cells and co-treatment with KP extract completely limited the overproduction of intracellular ROS.

These findings were also in accordance with the result of flow cytometric analysis with annexin V-FITC/PI staining, as our results showed that 5 mM of glutamate treatment for 14 h significantly increased the amount of both early and late apoptotic cells. This implied that most HT-22 cell death caused by glutamate occurred via the apoptotic pathway, which is in agreement with published data reported, which suggested that ROS is linked to necrosis at an early hour (12 h), and apoptosis is mainly activated at late hours (12–24 h), of glutamate exposure [29,51,52], and therefore glutamate-induced apoptosis is mediated via two mechanisms including a caspase-dependent pathway and a caspase-independent pathway. It has been reported that caspase-3 protein was not observed in HT-22 cells; therefore, glutamate should mediate cell death via the caspase-independent apoptosis pathway which involves calpain and AIF protein [51]. For this reason, we focused on the expression and translocation of AIF protein after glutamate treatment. The expressions of nuclear AIF protein and cytoplasmic AIF protein were compared in the presence or absence of KP extract, and the findings suggest that KP extract co-treatment recovered and prevented the apoptosis induction by glutamate. Therefore, AIF protein plays the crucial role in the glutamate-induced apoptosis cell death in HT-22 cells. 

Mitogen-activated protein kinases (MAPKs) are signal transduction molecules which are involved in cellular responses that lead to both cell death and survival [52]. In mammalian cells, MAPK families have been characterized into three groups, among which the MAPK/extracellular signal-regulated kinase (ERK) pathway, a member of MAPK families usually associated with cell survival [53], neurogenesis, and improving the survival rate of neurons in the hippocampus by activation of the MAPK pathway [54,55]. It has been known for many years that activation of ERK, is involved in BDNF-dependent survival effects [56]. On the basis of previous in vitro studies reported, glutamate could be mediated by c-Fos expression, which then induces BDNF expression [57,58]. BDNF is involved in low-level stimulation of NMDA receptors, which protect hippocampal neurons in culture against glutamate excitotoxicity by activation of the ERK pathway [59,60]. Similarly, BDNF is a representative neurotrophic factor associated with formation and storage of memory that activates cAMP-response element binding protein (CREB) through transducing cellular signaling involving PI3K, Akt, ERK1, ERK2, and CaMK [61,62,63]. On the basis of these previous results, the neuroprotective effect of KP extract may occur through BDNF expression and ERK pathway in HT-22 cells. Therefore, to support this hypothesis, we investigated the molecular mechanism of KP extract against glutamate excitotoxicity. Our results showed that BDNF expression decreased, and that Erk1/2 was activated in the presence of glutamate. By contrast, KP extract significantly diminished their phosphorylation induced by glutamate, and BDNF was also recovered to the control level. 

In this study, we also reported for the first time that KP extract had an anti-aging effect on *C. elegans*. Our results revealed that the mean and maximum lifespan of *C. elegans* were significantly increased after KP extract treatment, suggesting KP extract mediated anti-aging effects through extending the lifespan. For future research, the focus should be on the underlying biological mechanisms which are responsible for these effects. Moreover, we are looking forward to studying many aspects of KP in the future to elucidate its mechanisms of prevention and treatment of neurodegenerative disorders. It is noteworthy that the ability of KP extract and its phytochemicals to inhibit amyloid fibril formation, and reduce toxic levels of brain Aβ, as well as the potential to protect neuronal mitochondrial function in AD, should be further investigated.

## 5. Conclusions

Our results established the neuroprotective properties of KP extract on glutamate-induced cell death and also showed anti-aging effects through extending the lifespan of *C. elegans*, suggesting that KP extract may be a new candidate for both neuroprotectant and longevity-enhancing agent. 

## Figures and Tables

**Table 1:** Free radical scavenging activity. Total phenolic content and total flavonoid content of KP extract.

Sample	DPPH (% Inhibition)	ABTS (% Inhibition)	Phenolic Content (mg GAE/g Dry wt.)	Flavonoid Content (mg CEQ/g Dry wt.)
KP extract (1 mg/mL)	35.34 ± 1.71	54.89 ± 0.67	33.75 ± 2.18	52.25 ± 6.70

Values are mean ± SD (n = 3).

## Data Availability

Not applicable.

## Supplementary Materials

The following are available online at https://www.mdpi.com/article/10.3390/biology10040264/s1, Figure S1: The film Western blot images represent the expression level of Erk, pErk, BDNF, nuclear AIF, cytoplasmic AIF, Lamin B1 and β-actin, Figure S2: Identification and quantification of 5,7-DMF in KP extract by HPLC analysis, Figure S3: Neuroprotective effect of KP extract on glutamate-induced cytotoxicity in HT-22 cells, Figure S4: The effect of KP extract and glutamate on the viability of HT-22 cells, and Table S1: Results and statistical analysis of KP extract treated *C. elegans* lifespan assay.
