# Supplementary material for: Kaempferia parviflora Rhizome Extract Inhibits Glutamate-Induced Toxicity in HT-22 Mouse Hippocampal Neuronal Cells and Extends Longevity in Caenorhabditis elegans
Source: Biology (Basel). 2021 Mar 26;10(4):264. doi: 10.3390/biology10040264 (PMC8066628; doi:10.3390/biology10040264)
Supplement: Supplementary file 1 [file biology-10-00264-s001.pdf]

biology

MDPI

Supplementary materials

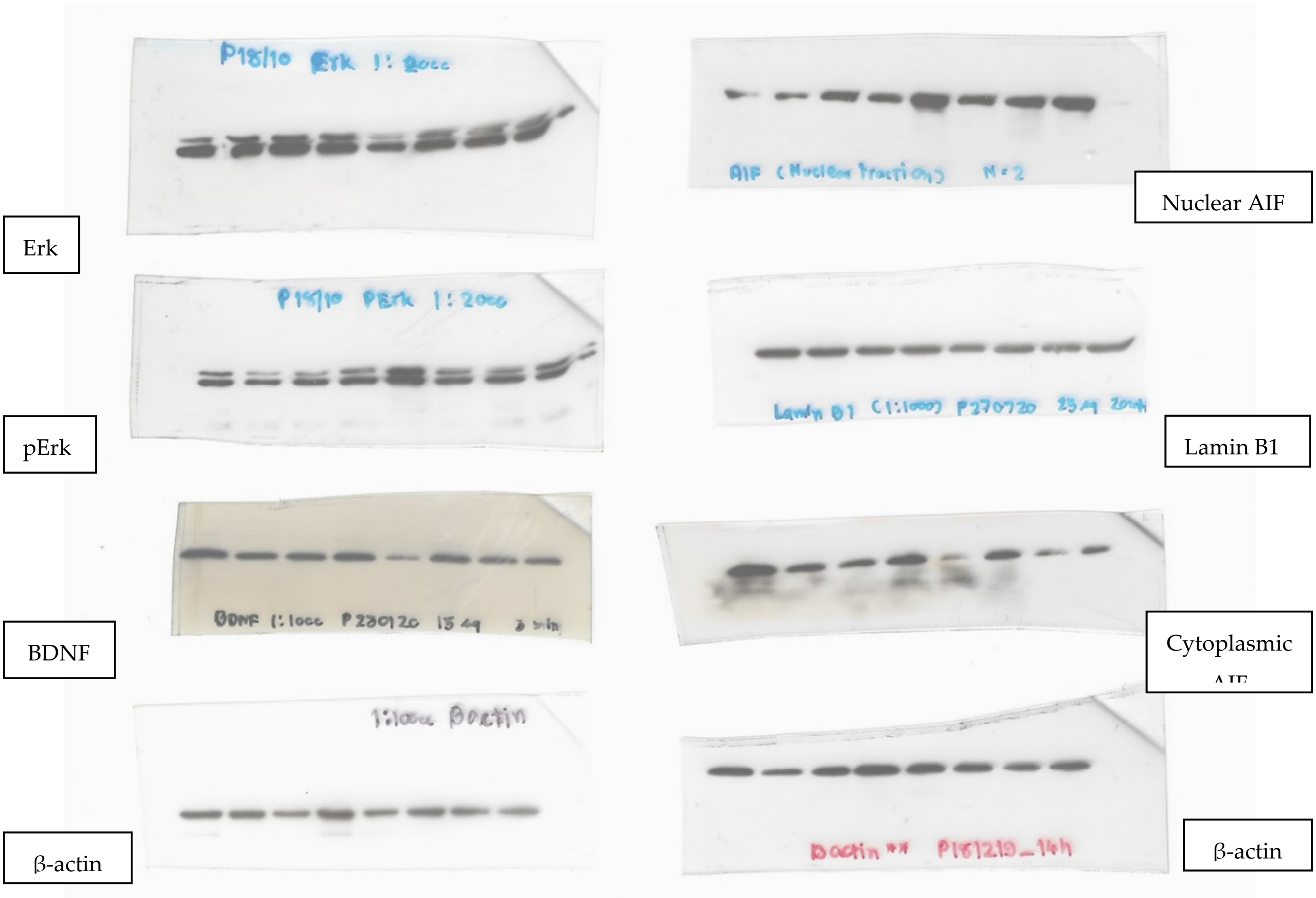

Figure S1: The film Western blot images represent the expression level of Erk, pErk, BDNF, β-actin (**Figure 4 of revised manuscript**) and nuclear AIF, Lamin B1, cytoplasmic AIF, β-actin (**Figure 5 of revised manuscript**).

In our present study, the content of 5,7-dimethoxyflavone (5,7-DMF) in *K. parviflora* rhizome extract was determined using the high-performance liquid chromatography (HPLC) analysis. The chromatogram showed the concentration of 5,7-DMF in KP extract (100 mg/mL) was 10037.32 ± 17.991 mg/L (10.04 ± 0.017 mg/mL) as shown in Figure S2. Therefore, the detection of 5,7-DMF in our KP extract as a major phytochemical certified the quality of our extract for this study. This finding was in line with other study since it has been known for a long time that 5,7-DMF is a major chemical constituent in KP rhizome extract.

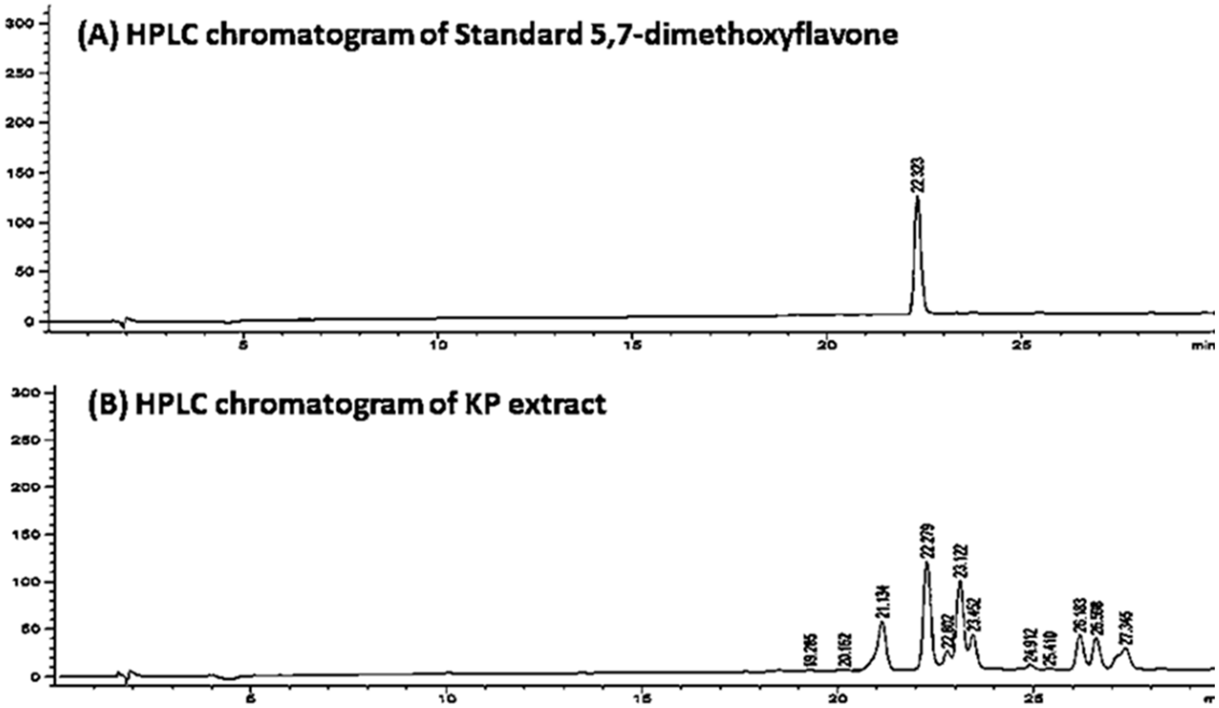

Figure S2: Identification and quantification of 5,7-DMF in KP extract by HPLC analysis. (**a**) Standard 5,7-DMF and (**b**) KP extract. Column nucleodure 100-5 C18ec (4.6 mm × 150 mm I.D., 5 µm); mobile phase methanol : 2% acetic acid in water, gradient elution; flow rate: 1 mL/min; injected sample 20 µL; detector : diode array detector at 280 nm.

This study was first in terms of testing the effect of KP extract on glutamate toxicity in HT-22 cells. Our preliminary study was designed to determine effects of pretreatment (the HT-22 cells were treated with KP extract for 12 h and then exposed to glutamate for 12 h) and co-treatment (HT-22 cells were simultaneously exposed to glutamate and treated with KP extract for 24 h). Results from the MTT assay showed that pre-treatment of cells for 12 h with different concentrations of KP extract did not lead to any significant protection, while in the co-treatment studies suggested a protective effect of the KP extract against cytotoxicity caused by glutamate (5mM). However, the HT-22 cells were stressed with 5 mM glutamate for 24 h resulting in a reduction of cell viability less than 50% which inappropriate for subsequent experiments. Therefore in this study the HT-22 cells were exposed to glutamate and treated with KP extract for 14 h.

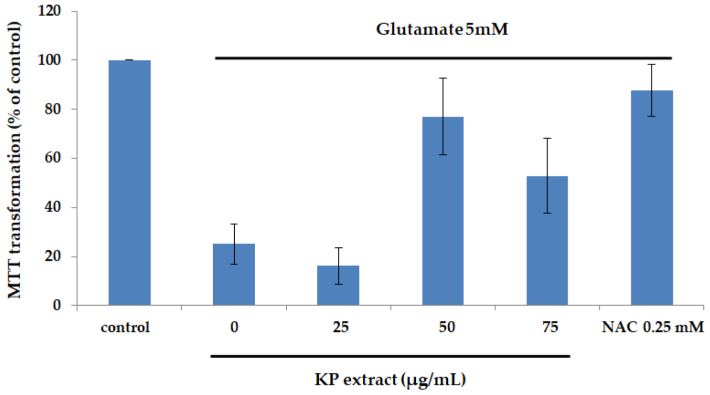

(**a**)

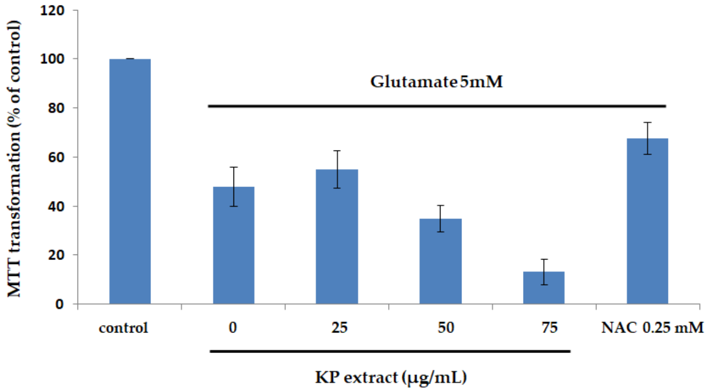

(**b**)

Figure S3: Neuroprotective effect of KP extract on glutamate-induced cytotoxicity in HT-22 cells. (**a**) Cells were exposed to 5 mM glutamate alone or glutamate in combination with different concentrations of KP extract for 24 h. (**b**) Cells were pretreated with various concentration of KP extract for 12 h and then exposed to 5 mM glutamate for 12 h. NAC (0.25 mM) was used as positive control. Cell viability was determined by MTT assay. Each bar represents the mean ± SEM from 2 independent experiments per group.

The N2 wild-type *C. elegans* cultured on various concentrations of KP extracts (100, 300, 500, 700 and 900 μg/mL) were monitored to determine whether KP extract effect on the lifespan of *C. elegans*. Our result revealed that the control group without KP extract had a mean life span of 13.86 ± 0.58 days. There were no statistically significant different in the life span of *C. elegans* control group and those exposed to KP extract at lower concentration (100 and 300 μg/mL). But *C. elegans* treated with KP extract at 500 and 700 μg/mL were significantly extended the mean of life span to 17.24 ± 0.55 ($p<0.001$) and 16.28 ± 0.42 days ($p<0.05$), respectively (Table 3 and Figure 9).

Table S1: Results and statistical analysis of KP extract treated *C. elegans* lifespan assay.

Treatment	No. of worms	Mean lifespan (days)	Percentage of increased mean lifespan (vs. control)	P value vs. control
Control	50	13.86 ± 0.58	-	-
KP 100 μg/mL	50	14.42 ± 0.37	4.04	0.984
KP 300 μg/mL	50	15.14 ± 0.42	9.24	0.553
KP 500 μg/mL	50	17.24 ± 0.55	24.39	0.001
KP 700 μg/mL	50	16.28 ± 0.42	17.46	0.028
KP 900 μg/mL	50	14.02 ± 0.43	1.15	1.000

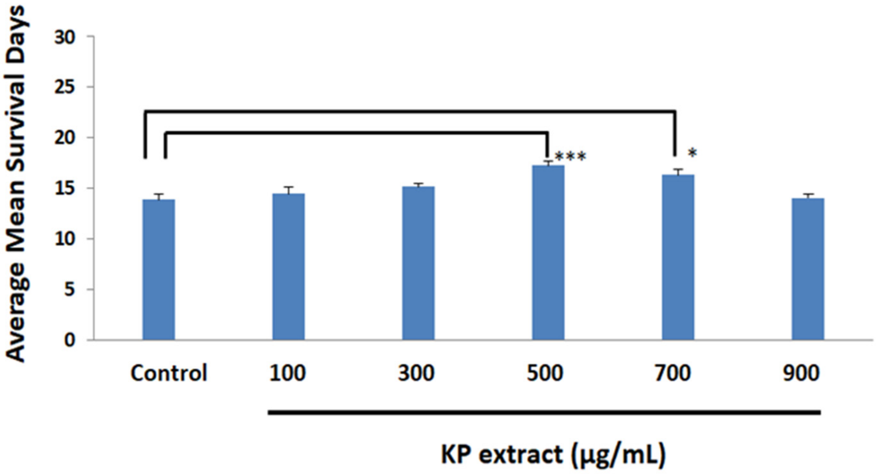

Figure S4: Effect of KP extract on the lifespan of N2 wild-type *C. elegans*. The L4 larval stages nematodes were treated at 15 ^{0}C with various concentration of KP extracts (0, 100, 300, 500, 700 and 900 μg/mL). The survival was counted starting from day 1 of adulthood to death. The bars represent the mean lifespan when treated with KP extract. The experiments were performed in 5 experiment trials. and shown as the mean ± SEM, *$P < 0.05$ and ***$P < 0.001$ vs. control group.
